# Assessment of ‘Cabernet Sauvignon’ Grape Quality Half-Véraison to Maturity for Grapevines Grown in Different Regions

**DOI:** 10.3390/ijms24054670

**Published:** 2023-02-28

**Authors:** Yanhua Ren, Ehsan Sadeghnezhad, Xiangpeng Leng, Dan Pei, Tianyu Dong, Peian Zhang, Peijie Gong, Haifeng Jia, Jinggui Fang

**Affiliations:** 1 Key Laboratory of Genetics and Fruit Development, Horticultural College, Nanjing Agricultural University, Nanjing 210095, China; 2 Horticultural College, Qingdao Agricultural University, Qingdao 266109, China

**Keywords:** Cabernet Sauvignon, half-véraison, transcriptome, berry quality, regions

## Abstract

Grapes are widely cultivated around the world and their quality has distinct regional characteristics. In this study, the qualitative characteristics of the ‘Cabernet Sauvignon’ grape variety in seven regions, from half-véraison to maturity, were analyzed comprehensively at physiological and transcriptional levels. The results indicated that the quality traits of ‘Cabernet Sauvignon’ grapes in different regions were significantly different with obvious regionality. Total phenols, anthocyanins, and titratable acids were the main factors of the regionality of berry quality, which were very sensitive to changes in the environment. It should be noted that the changes in titrating acids and total anthocyanin of berries vary greatly from half-véraison to maturity between regions. Moreover, the transcriptional analysis showed that the co-expressed genes between regions characterized the core transcriptome of berry development, while the unique genes of each region reflected the regionality of berries. The differentially expressed genes (DEGs) between half-véraison and maturity can be used to demonstrate that the environment of the regions could promote or inhibit gene expression. The functional enrichment suggested that these DEGs help to understand the interpretation of the plasticity of the quality composition of grapes according to the environment. Taken together, the information generated by this study could contribute to the development of viticultural practices aimed at making better use of native varieties for the development of wines with regional characteristics.

## 1. Introduction

*Vitis vinifera* is the most economically valuable fruit tree in the world, which is widely used in the food and beverage industries [1]. Grapes have phenotypic plasticity, which allows them to adapt to climate change and produce different qualities of drinks of the same variety [2,3]. ‘Cabernet Sauvignon’ is a red grape known for its thick and durable skin. It is also rich in flavor and has a high tannin content, making it a perfect partner for foods such as rich grilled meats, pepper sauces, and dishes with a high flavor [4]. Meanwhile, the uniqueness of berry quality depends on the interaction between the vines, environmental factors, and cultural practices so that wines exhibit distinct ‘terroir’ characteristics in different environments [5]. 

Experimental evidence demonstrated that berry development is a complex process and environmental factors such as temperature, humidity, water, and altitude had a profound influence on berry quality formation, giving it a metabolic model with regional characteristics [6,7,8,9]. For instance, temperature and light intensity could affect anthocyanins and phenols synthesis [9,10,11,12]. Insufficient water or drought stress could increase the anthocyanin content of berries and reduce fruit growth [13]. In addition, high temperature induces an enzyme reaction in sugar biosynthesis, which increases sugar content and decreases acidity [14]. The total acidity of grapes is mainly determined by the amount of tartaric acid and malic acid, and the reduction in acidity is mainly due to the reduction in malic acid content [15]. Elevated temperatures (>35 °C) increase the tannins concentration in berries [16].

Different regions with different climates possess a variety of environmental factors which may influence gene expression in grape berries. There were major differences in the amount and quality of expressed genes of ‘Cabernet Sauvignon’ in the regions in response to environmental stimuli [17]. It was found that the up-regulated genes were mainly involved in light and growth hormone signaling responses, polyphenol metabolism, terpene aroma synthesis, and other processes in Bordeaux. In Nevada, environmental and geographical influences led to water stress response, cold stress response, and ABA signaling, which affected the characteristics of grape quality traits and industrial processing [11,17,18].

During berry development, the process of the color change of grapes is called véraison, which is accompanied by changes in physical and chemical factors, both intracellular and intercellular, and continues to maturity [19]. This stage is accompanied by the regulation and accumulation of sugars, acids, polyphenols, and anthocyanins. Therefore, it is the critical stage for influencing berry quality [20]. The synthesis and accumulation of berry-quality metabolites affect the various berry characteristics during the growth and development processes. Acids and tannins are mostly synthesized at an early stage, and their concentrations decrease when the berries mature. Sugars and anthocyanins accumulate rapidly at the early stage of maturation. The variability in the synthesis of these primary substances leads to a complex plasticity of berry quality [21]. Therefore, analysis of trait changes from half-véraison to maturity is useful to understand the nature of quality formation. 

In this study, the changes in grape berry quality from half-véraison to maturity in different regions of Shandong were analyzed extensively, and the influence of the regional environment on the formation of berry metabolites was discussed. In addition, RNA-seq is used to monitor the changes in grape quality in the regional environment, thereby deepening the understanding of grape quality regionality. This study can provide a new theoretical basis for the regional analysis of the berry quality of ‘Cabernet Sauvignon’ grapes and provide a reference for the practice of directional viticulture. 

## 2. Results

### 2.1. Characteristics of Different Regions

The wine-grape-growing regions of China display unique ecological conditions either from south to north or from east to west. In this work, the seven regions including Dezhou (DZ), Laixi (LX), Penglai (PL), Rizhao (RZ), Rushan (RS), Tai’an (TA), and Zaozhuang (ZZ) were in Shandong province, China (Figure S1). The environmental conditions of those regions were distinctly different. Among them, the average annual temperature ranged from 13.39 to 15.69 °C, precipitation ranged from 747.33 to 1376.91 mm, the annual sunshine hours ranged from 1552.8 to 1694.17, the relative humidity ranged from 56.15 to 85.8% (Table S1). Pearson’s correlation analysis showed that environmental conditions significantly affected grape quality characteristics across regions (Figure S2). 

### 2.2. Quality Characteristics of ‘Cabernet Sauvignon’

**Figure 1:**
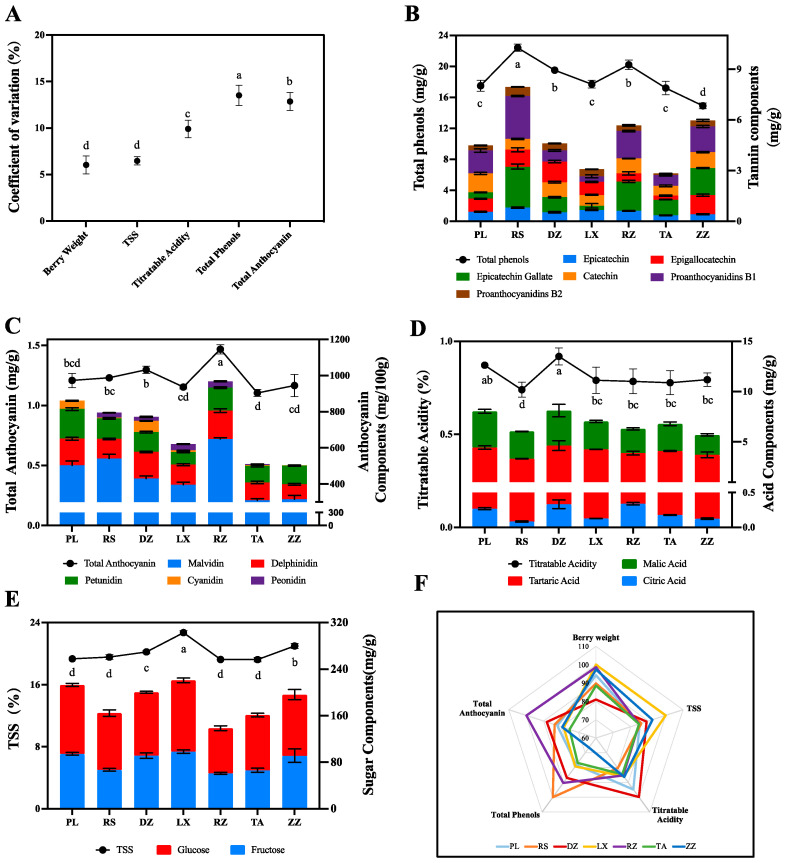
Quality characterization of ‘Cabernet Sauvignon’ berries in seven regions including DZ, LX, PL, RZ, RS, TA, and ZZ at maturity. (**A**) Measurement of Coefficient of Variation (C.V) related to berry weight, TSS, titratable acidity, total phenols, and total anthocyanins of ‘Cabernet Sauvignon’ at maturity level. (**B**–**E**) Analysis of main components related to total phenolics, total anthocyanins, titratable acid, and TSS, respectively. The broken line represents the quality traits in different regions. The bar represents the charge ratio in different regions from half-véraison to maturity. The different letters indicate the significant differences between regions (*p* ≤ 0.05) using Duncan’s test. Experiments were performed using three replicates. mg/g refers to fresh weight. (**F**) Evaluation of seven regions using radar map according to quality characterization.

The sugar-acid ratios of berries in 7 regions were above 22, which was an important indicator of grape maturity (Table S2). Berry weight, total soluble solids (TSS), titratable acidity, total phenols, and anthocyanins varied significantly across regions (Figure 1A). Among them, the coefficient of variation (C.V) for total phenols was the highest at 13.51%, varying from 14.88 to 22.40 mg/g (Figure 1A,B). The anthocyanin content of berries ranged from 1.10 to 1.47 mg/g with C.V at 12.85%, showing obvious variations between regions (Figure 1A,C). Titratable acids also varied in the range of 0.74 to 0.92% with a C.V of 9.91% (Figure 1A,D). TSS and berry weight showed relatively little variation across regions, with C.V at 6.47% and 6.04%, respectively. TSS of berries in the regions ranged from 19.22 to 22.70% (Figure 1A,E). Meanwhile, berry weight ranged from 1.84 to 2.08 g (Figure 1A). Radar plots were also used to characterize the quality of ‘Cabernet Sauvignon’ between regions. As shown in Figure 1B, the berries from LX were high on TSS, the berries from DZ and PL were high on titratable acid, the berries from RS and RZ were high in total phenols, whereas the berries from RZ were rich in anthocyanins. 

In detail, an analysis of the main components of sugars, acids, anthocyanins, and phenolics in the berries from various regions showed that the content of each component was variable. For example, the berries in LX regions were rich in TSS, and the accumulation of glucose and fructose indicated a significant difference from other regions (Figure 1E). The berries from DZ and PL were rich in titratable acids, with 0.92% and 0.87%, respectively. Meanwhile, the content of the main organic acids in wine grapes including tartaric acid and malic acid significantly were higher in DZ and PL samples than in the others (Figure 1D). Five main anthocyanin components were determined and found that malvidin was the dominant anthocyanin component with the highest content of 648.77 mg/100g in RZ samples and the lowest content of 309.92 mg/100 g in TA samples. In addition, peonidin and cyanidin were the most variable anthocyanin components in the seven regions, with variance multipliers of 159.02-fold and 105.11-fold, respectively. Differently, delphinidin and petunidin showed less variation across all regions such as malvidin. Among the seven regions, the highest content of delphinidin was 157.47 mg/100 g in RZ, while the highest content of petunidin was 164.08.mg/100 g in PL (Figure 1C, Table S3). Phenols contents were also variable with the highest content in RS samples (22.40 mg/g) and the lowest in LX samples (14.88 mg/g). Of the six major phenolic components identified, epigallocatechin and epicatechin gallate contents were detected at 3.37 and 3.32 mg/g, respectively, in berries of RS. Meanwhile, the main phenolics from DZ and ZZ were epigallocatechin (up to 2.98 mg/g) and epigallocatechin gallate (up to 3.17 mg/g) (Figure 1B, Table S3).

### 2.3. Different Regions Affect the Quality of Grapes from Half-Véraison to Maturity

**Figure 2:**
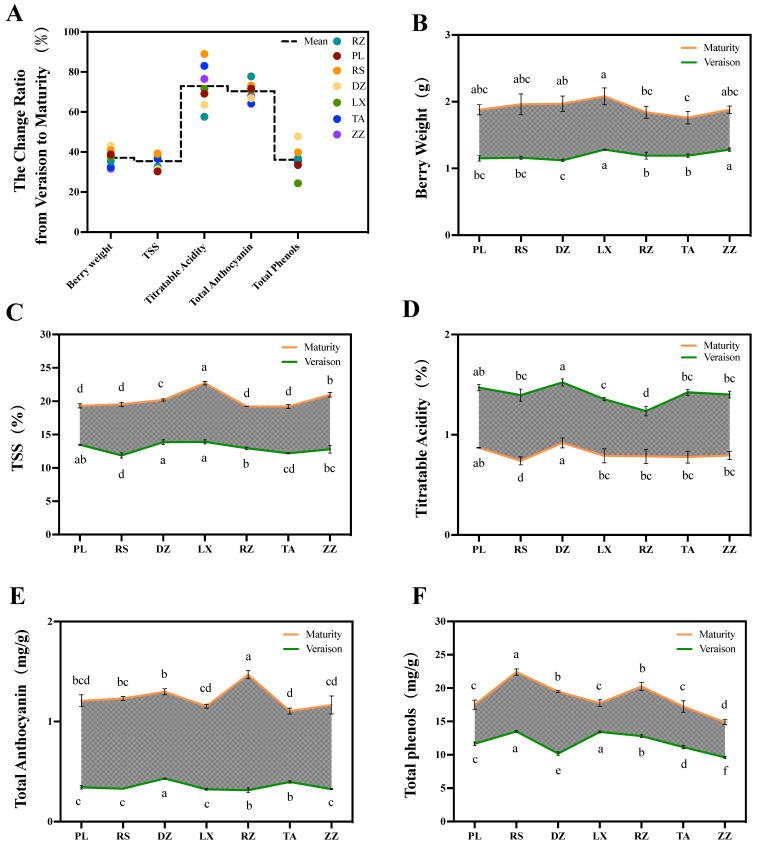
The change ratio of five quality indices during half-véraison and maturity stages in grape berries belonging to different regions. Variance ratio (**A**) and changes of five quality indices including berry weight (**B**), TSS (**C**), titratable acids (**D**), anthocyanins (**E**), and total phenolics (**F**) between half-véraison and maturity stages of grape berries. The broken line represents the quality indices in seven regions including DZ, LX, PL, RZ, RS, TA, and ZZ. The green and orange lines represent the change in different regions in the half-véraison and maturity stages. The different letters indicate the significant differences between regions (*p* ≤ 0.05) using Duncan’s test. Experiments were performed using three replicates.

Half-véraison is the important intermediate process of the maturation phase of grapes, which leads to drastic physical and chemical changes in berries. In this study, five quality indices were studied including berry weight, TSS, titratable acids, anthocyanins, and total phenols between half-véraison and maturity of grape berries. Of these, titratable acids and anthocyanins were more variable, with a variance ratio of approximately 72.91% and 70.36%. The variation in total phenols, TSS, and berry weight was relatively less, with a variance ratio from 35.42 to 37.11% (Figure 2A). Furthermore, the change ratio in quality indices between regions depended on the environmental conditions and climate (Figure 2B–F). Overall, the pattern of change ratio of TSS was similar from one region to another. For example, the change ratio of TSS in RS, LX, and ZZ regions had a high correlation in both half-véraison and maturity stages (Figure 2A,C). Meanwhile, a similar trend was observed for berry weight. The extent of variation in PL, RS, LX, and DZ was a high correlation with berry weight at maturity (Figure 2A,B). For titratable acids, the change ratio ranged from 57.59% in RZ to 88.98% in RS. Therefore, the content of titratable acids in regions of RS and RZ was significantly lower at maturity and half-véraison stages, respectively (Figure 2A,D). Total anthocyanins were also changed from 64.18% in TA to 77.73% in RS. Interestingly, at the maturity and half-véraison stages, the anthocyanin accumulated at the maximum levels in regions of RZ and DZ, respectively, but the region of TA indicated the lowest content at the maturity stage among all regions (Figure 2A,E). The change ratio of total phenols varied considerably between regions, ranging from 24.34 in LX to 47.77% in DZ. In particular, the total phenolic content in regions of LX and RS significantly increased at half-véraison, while berries belonging to the ZZ region indicated the lowest content at both maturity and half-véraison (Figure 2A,F). 

### 2.4. Transcriptome Analysis Overview

Transcriptomes of ‘Cabernet Sauvignon’ in seven regions of half-véraison and maturity were sequenced. In total, 177.82 Gb of raw data were obtained, and the number of reads varied from 39.80 to 46.15 million for 91.46–97.54% of accounting data. It provided adequately for the next expression profiling analysis, which could be used for the subsequent quantitative analysis of gene expression (Table S4). PCA also demonstrated reproducibility and variability among regions (Figure S3), verified the accuracy and reproducibility of the RNA-seq data, then randomly selected 17 genes involved in the different biological pathways for qPCR (Figure S4). The results of qRT-PCR had a good linear relationship with the transcriptome data, with a correlation coefficient (R2) of 0.90, which confirmed the reliability and accuracy of the data.

**Figure 3:**
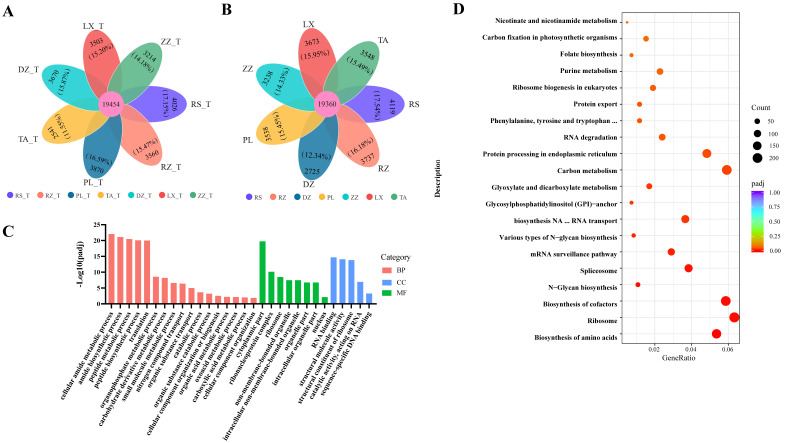
Petal mapping and KEGG pathway analysis for the overlapping and unique genes related to the localized samples in seven regions. (**A**,**B**) Petal map shows the overlapping and unique genes from regions at half-véraison (**A**) and maturity (**B**), in this figure ‘T’ means half-véraison stage. The percentage was the ratio of unique genes to the total genes in the region. (**C**) Category of biological functions related to DEGs according to the comparative combinations of different regions at maturity. (**D**) The distribution of sample data by stacking dots along the horizontal axis to represent the frequencies of different values. Abscises represent the GeneRatio, and the ordinate represents in terms of KEGG pathway. The GeneRatio refers to the ratio of the sample number to the background enriched in the pathway. Padj indicated that those closer to zero express greater enrichment.

The number of genes detected during berry development (from half-véraison to maturity) varied substantially in this study. At half-véraison, the highest and lowest number of genes were in the collected samples of RS (23,480 genes) and TA (21,995 genes), respectively. At maturity, the lowest was 22,085 belonging to the DZ region, and the highest was 23,479 belonging to the RS region. The petal map indicated that about 85% of the genes were co-expressed and about 15% of the unique genes across regions (Figure 3A,B). Assessing the function of co-expressed genes among regions, the co-expressed genes were enriched for a total of 916 biological functional groups and 119 metabolic pathways (Tables S5 and S6). The major cellular processes including cytoplasmic, nuclear, small molecule metabolism cytosolic amide metabolism, peptide metabolism, organic acid metabolism, and other biological functions, had been significantly enriched and involved numerous genes (padj ≤ 0.05) (Figure 3C and Figure S5A). Additionally, many co-expressed genes had been significantly enriched into metabolic pathways such as the ribosome, carbon metabolism, amino acid biosynthesis, and cofactor biosynthesis (Figure 3D and Figure S5B), which likely contributed to the growth and development of grapes.

### 2.5. Cluster Analysis of Transcriptomic Data at Maturity

**Figure 4:**
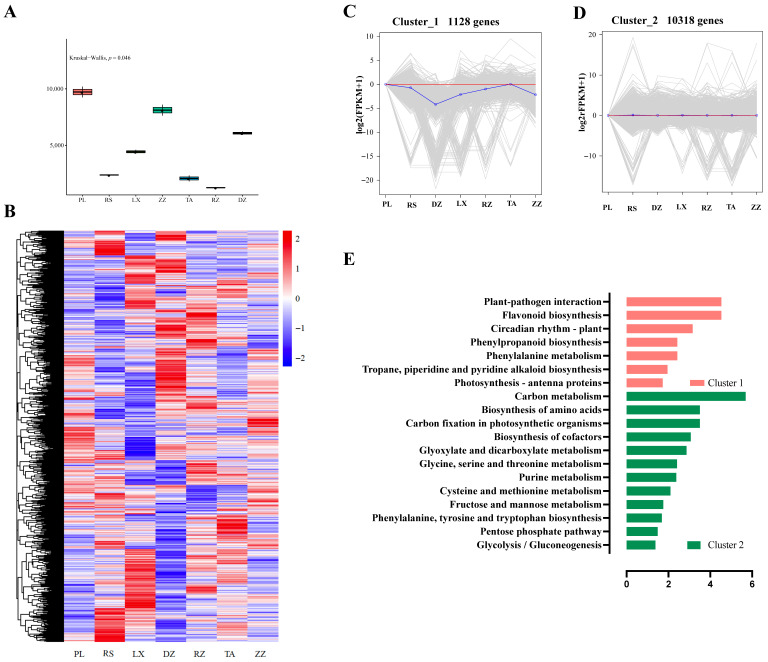
Classification of transcriptomic data of the localized grape berries at maturity in different regions. Seven regions were included DZ, LX, PL, RZ, RS, TA, and ZZ. (**A**) Kruskal-Wallis test at maturity. (**B**) Heatmap analysis using FPKM values of genes in different regions at maturity. (**C**,**D**) K_means clustering analysis of 11,783 genes into two groups. (**E**) Histogram showing the involvement of metabolic pathways of the classified genes in Cluster1 and Cluster2 at maturity. The abscissas represent the GeneRatio, and ordinate represents KEGG pathway. The GeneRatio refers to the ratio of the sample number to the background number enriched in the pathway.

The Kruskal-Wallis test indicated that the berry transcriptions from different regions were very different at maturity (Figure 4A). K_means clustering analysis of the resulting 11,783 genes, whose expression profiles at maturity showed a significant difference in modulation in at least one region (Figure 4B), revealed two major clusters (Figure 4C,D). Cluster 1 included 1128 genes, which were primarily involved in flavonoid biosynthesis, circadian rhythm, phenylpropane synthesis, and other processes (Figure 4C,E). The differential expression of these genes contributed to regional effects on berry quality. Cluster 2 included 10,318 genes, which are mainly involved in carbon metabolism, amino acid biosynthesis, glycolysis, glyoxylate metabolism, and other pathways (Figure 4D,E). These data implied that significant changes in the expression of genes were influenced by regional characteristics. 

### 2.6. Gene Ontology and KEGG Enrichment Analysis of DGEs

**Figure 5:**
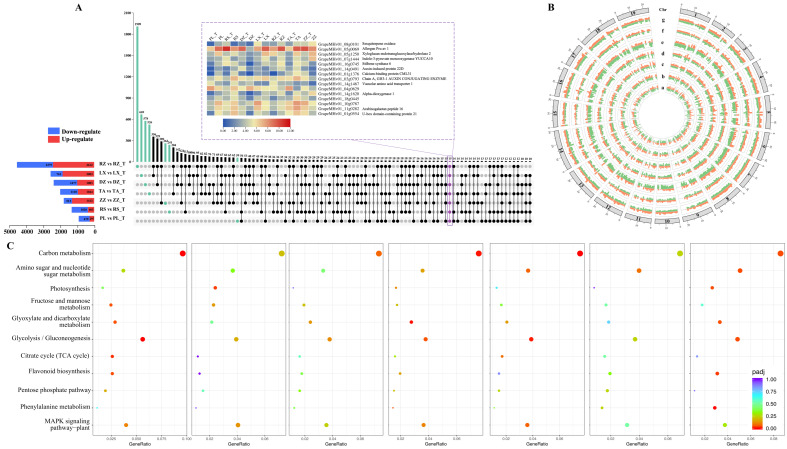
Determination of up- and down-regulated genes among the collected samples from seven regions. (**A**) Up-set showing the overlapping and unique genes of different regions, the blue column depicts down-regulate genes, the red column depicts up-regulate genes, and the heatmap represents DEGs based on FPKM (fragments of transcripts per million fragments mapped) value. (**B**) Circos diagram demonstrates the pattern of DEGs in different regions, which are labeled from a to g. The 19 chromosomes are shown by the first outer ring. The seven concentric rings from outside to inside represent the corresponding seven regions in this figure. Orange color in each cycle represents DEGs based on FPKM value at maturity, while green color represents DEGs at half-véraison. (**C**) Plot shows the degree of enrichment of KEGG metabolic pathways in different regions. The abscissas represent the GeneRatio, and the ordinate represents KEGG pathway. The GeneRatio refers to the ratio of the sample number to the background number enriched in the pathway. The letters a–g called for PL vs PL_T, RS vs RS_T, ZZ vs ZZ_T, TA vs TA_T, DZ vs DZ_T, LX vs LX_T, and RZ vs RZ_T, respectively. In this figure, T means half-véraison.

Up-set plots indicated that the DEGs varied widely across regions, ranging from 976 in PL to 4891 in RZ. Furthermore, DEGs had different expression patterns among regions. For example, the RZ, LX, and ZZ regions possess a higher number of up-regulated DEGs than down-regulated genes, while it was vice versa in DZ, PL, RS, and TA. Interestingly, the regions with high multiple-quality traits tended to have a higher number of up-regulated genes (Figure 5A). In addition, 15 genes of these DEGs were differentially expressed in all regions, involved sesquiterpene oxidase, xyloglucan endotransglucosylase, indole-3-pyruvate monooxygenase, stilbene synthase 6, auxin, and calcium-binding protein. Circos plots visualize the expression patterns of DEGs, the FPKM (Fragments per kilobase of transcript per million mapped fragments) as a gene expression unit at maturity and half-véraison stages for DEGs. As shown in Figure 5B, the expression trends of DEGs were significant differences across regions and developmental stages. The expression patterns of DEGs suggested these geographic differences in regions could inhibit and promote many gene expressions. Furthermore, DEGs were mainly located on chromosomes 18, 7, 14, 1, 4, 5, 6, and 8, with the highest number of DEGs on chromosome 18 (Figure S6). Moreover, the highly expressed genes were mainly distributed on chromosomes 5, 18, 7, 8, 1, 14, 6, and 4, indicating that key genes affecting berry development and quality formation were mainly distributed on these chromosomes.

The biological functions of DEGs were assessed based on gene ontology (GO) (classified molecular function, cellular component, and biological process). The DEGs were enriched into 843 categories of biological functions, containing 434 biological processes, 114 cellular components, and 295 molecular functions (Table S6). Among these, the metabolic process of the small molecule metabolic process, carbohydrate metabolic process, transcriptional regulatory activity, and DNA binding transcription factor activity were annotated with high enrichment scores. Some of the top GO categories included nicotinamide nucleotide, carbohydrate catabolism, carboxylic acid biosynthesis, acetone acid, glycolysis process, organic acid, phosphoribosyl sugar biosynthesis process, and glutamine family amino acid metabolic process were significantly enriched in multiple regions (Figure S7).

The DEGs between half-véraison and maturity from different regions were mapped to the Kyoto Encyclopedia of Genes and Genomes (KEGG) to obtain 115 metabolic pathways (Table S8). Some of the top KEGG metabolism pathways included carbon metabolism, biosynthesis of amino acids, biosynthesis of cofactors, plant hormone signal transduction, plant–pathogen interactions, ribosome, glycolysis/gluconeogenesis, and MAPK signaling pathway–plant. In addition, different regions prioritize the major metabolic pathways involved in the berry quality formation, which were 11 important pathways (Figure 5C). For example, the production of flavonoids in the collected samples of PL and ZZ regions indicated 19 and 14 DEGs that were significantly enriched in the flavonoid biosynthesis pathway. In LX, PL, RZ, and ZZ regions, 52, 41, 66, and 22 DEGs were significantly enriched in glycolysis/gluconeogenesis, respectively. In addition, the citric acid cycle (TCA cycle) and glyoxylate and dicarboxylic acid metabolism pathways also reached significant enrichment in the PL region. Phenylalanine metabolism and glyoxylate and dicarboxylate metabolism were significantly enriched in LX and ZZ regions.

## 3. Discussion

### 3.1. Evaluation of Berry Quality Dependence on the Region Characteristics

**Figure 6:**
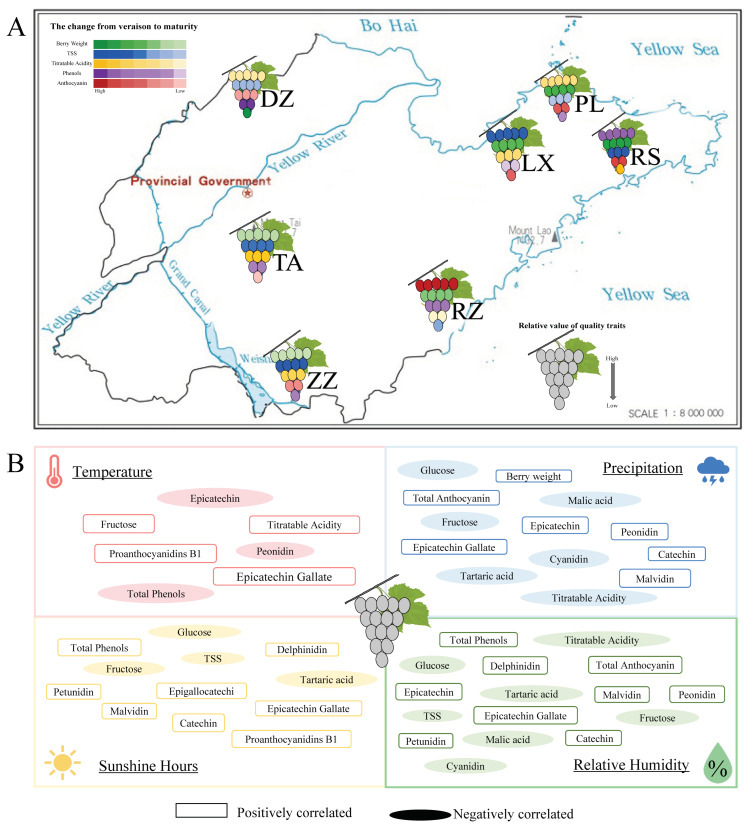
(**A**) Models of berry quality characteristics of ‘Cabernet Sauvignon’ in different regions and their forming from coloration to maturity. Green represents berry weight, blue represents TSS, yellow represents titratable acids, purple represents phenols, and red represents anthocyanins. The shades of color were used to characterize the change from half-véraison to maturity. The number of berries per color represents the relative value of quality traits. (**B**) Effects of environmental factors on grape quality. Boxes represented grape qualities positively regulated by environmental conditions; ovals represented grape qualities negatively regulated by environmental conditions.

It is common knowledge that the interaction between the environment and plants is the main factor in the quality of the grape. Environmental change plays a crucial role in quality formation [22,23,24]. Shandong as a production area has a variety of terrains, such as mountains, plains, and hills, which cause climate change. Meanwhile, the coastline of over 3000 m makes the difference in temperature in different regions. Consequently, ‘Cabernet Sauvignon’ from Shandong province is produced with different qualities and exhibited clear regional characteristics (Figure 6A).

The quality of berries is mainly on sugars, organic acids, and secondary metabolites such as polyphenols and anthocyanins [25]. Multivariate analysis indicated that phenolics, minerals, amino acids, and other components could be used to characterize and distinguish between regions [26,27,28]. Therefore, active compounds can be used as distinguishing markers. For example, terpenes and phenylalanine derivatives in Xinjiang wine were used as markers to distinguish different beverages between the north and south [4]. In this study, phenolic and anthocyanins were the main factors affecting the berry quality in Shandong-producing areas (Figure 1A). This is explained by the sensitivity of phenolic biosynthesis in berries to environmental factors [29]. In this study, total phenols were positively correlated with latitude and longitude, sunshine hours, and relative humidity of regions, and negatively correlated with temperature. Anthocyanins were positively correlated with relative humidity, precipitation, and sunshine hours (Figure 6B and Figure S2). Light and diurnal temperatures were able to inhibit or activate the expression of genes associated with phenolic and anthocyanin metabolism pathways, thereby significantly altering metabolite content [30]. Furthermore, a study on ‘Cabernet Sauvignon’ from five wine-producing regions in Chinese showed that similar terroirs had similar phenolic compositions [31]. In this study, six polyphenol components were primarily detected, although the difference in the ecological environment and microclimate led to large differences in the content of polyphenol components. Of these, the RS region is mainly characterized by epigallocatechin and epigallocatechin gallate, which could be due to the abundant sunshine in RS region (Figure 6B and Figure S2), while the DZ region was characterized by epigallocatechin. Berries from the ZZ region were like California wine [32] with epigallocatechin gallate as a phenolic compound (Figure 1B, Table S3). 

Also, malvidin was the main anthocyanin component detected in this study (Figure 1C), moreover, and the malvidin content of berries in RZ was significantly higher than that in other regions. This could be related to the unique geographic location located between the mountains and the ocean and the unique ecology that had high diurnal temperature difference, precipitation, and relative humidity of RZ (Figure 6B and Figure S2). In previous studies, grapes grown in large diurnal amplitude climates had higher sugar concentrations and photosynthetic rates; therefore, they had significantly improved fruit color and taste [33,34,35]. During grape maturation, the content of sugars, amino acids, and phenols increased, while organic acids decreased [36,37]. The acidity of grape berries depends on tartaric acid and malic acid levels, and studies have shown that higher temperatures effectively affect the balance between the synthesis and degradation of organic acids [15,26,38]. In this study, differences between constituents of organic acid in regions may be related to temperature during growth (Figure 1D). This hypothesis must be confirmed by subsequent experiments. Sugar is a key regulator of several primary and secondary metabolic pathways in response to environmental stimuli in the vineyard as well as abiotic stress [39]. In this study, berries from LX significantly accumulated TSS, glucose, and fructose more than in other regions (Figure 1E). Additionally, TSS concentrations increased in ‘Cabernet Sauvignon’ berries exposed to the sun when compared to shadow. It demonstrated that light and vine moisture status could affect TSS [40]. 

### 3.2. Key Period from Half-Véraison to Maturity for Berry Quality Formation 

In this study, sugar, acid, anthocyanins, phenols, and other traits had a significant change from the half-véraison to maturity. Since the growth and development of grapes follow a double sigmoidal curve, the characteristic of rapid change corresponded to an S-shaped growth curve. The first phase of the curve is mainly manifested by an increase in berry size and weight, and the second phase of the curve is mainly manifested by an increase in fruit sugar, anthocyanin, and other quality traits that are defined as maturity. While veraison is the beginning of the second phase curve, characterized by the beginning of coloring and softening of the berries, rapid accumulation of sugars, and degradation of acids [19,23]. In this study, titratable acids and anthocyanins had a higher rate of change, approximately twice as high as total phenol, TSS, and berry weight (Figure 2A). Plant secondary metabolism produces different compounds in response to environmental stimuli [39]. In addition, the spatiotemporal nature of the synthesis and accumulation of berry-quality compounds makes it possible to create complex plasticity of grapes [20,41].

As the berries mature, the titratable acid decreases dramatically. The rate of change from half-véraison to maturity in RS regions was as high as 88.98% (Figure 2A,D). Anthocyanin is another quality trait with a high rate of change. Additionally, there was a high correlation between the rate of change from half-véraison to maturity and the anthocyanin content at maturity, such as TA and RZ regions (Figure 2A,E). Furthermore, this result provided evidence for the rapid accumulation of anthocyanin after half-véraison. The change ratio of total phenols varied considerably between regions, with the highest in DZ being approximately twice in LX. It was noteworthy that the total phenols content from LX was significantly higher than that from DZ at half-véraison, while the opposite profile was observed at maturity (Figure 2A,F). It could be linked to the complex and variable climate type of the Shandong production area. Previous studies showed that sufficient light and cold weather contributes to the accumulation of some phenolic substances [42]. The variation in TSS was similar in different regions, where the change ratio from RS, LX, and ZZ regions had a high correlation with the content of the two stages (Figure 2A,C). In this study, the high sugar from LX, ZZ, and DZ regions and the rapid rise in these two stages may be related to their location inland. These regions were characterized by a temperate monsoon climate with high average temperatures (Table S1). Studies have shown that high temperatures increase photosynthesis, accelerate metabolism, and promote sugar biosynthesis and transport [43]. A similar pattern was observed for berry weight. The berry weight at maturity was closely related to the rate of change in the PL, RS, LX, and DZ regions (Figure 2A,B).

### 3.3. Transcriptome Analysis of Quality Characteristics of Berries during Half-Véraison and Maturity

Rapid advances in the sequencing of grapevine reference genomes and the development of new tools for transcriptomic data have contributed to recent advances in the analysis of dynamic gene expression during berry development [44,45,46]. Recently, researchers used omics approaches and determined approximately 18% of berry transcripts changed in response to the environment [24]. A deep transcriptomic shift was found at Corvina to drive the berry through the maturation program [47]. Data from this study indicated that the berry quality has responded to multiple potential environmental factors in seven regions, thus demonstrating the evident regionality. Transcription abundance and functional annotation of genes provided important clues to the factors affecting berry quality. More experiments are also necessary to ensure the follow-up of these observations.

The abundance of multiple genes in all regions could be used as a useful lever to adjust berry quality [22]. Based on the current dataset, berries from different regions show differ significantly at maturity (Figure 4A). Furthermore, K_means cluster analysis to determine differences in molecular levels. The genes classified in cluster1 were primarily involved in flavonoid biosynthesis, circadian rhythm, phenylpropane synthesis, etc. These genes pointed to a regional expression of berries (Figure 4C,E). Previous studies have investigated the pathway of phenylpropanoids, one of the most environmentally dependent metabolic constituents, with a favorable correlation between metabolite levels and induction of gene expression [3]. The genes in Cluster 2 were mostly involved in the carbon metabolism, amino acid biosynthesis, glycolysis, and glyoxylate metabolism pathways (Figure 4D,E). These genes highlighted deep metabolic differences among regions. Therefore, the genes involved in forming differences in berry quality may serve as indicators. 

Current data suggested that the amount and expression of DEGs varied greatly among regions (Figure 5A,B). This demonstrated that those differences in environmental conditions affect the development of berry quality. As with previous results, transcriptome data representing berries may be an important tool for assessing the specificity of berry quality [48]. In this study, 15 DEGs between half-véraison and maturity were expressed differently in the seven regions studied. These genes serve as good candidates for markers of berry development and regionality quality (Figure 5A). A broader range of studies from more regions and environments is needed to identify the genes involved in berry and quality development. The specific environmental conditions will be reflected in the regulation of certain metabolic pathways in berries. Bright light, hypothermia, and dehydration can affect the expression of genes in primary and secondary metabolic pathways and the accumulation of metabolites [34,35,49,50,51]. Here investigated the changes in the abundance of the gene that may contribute to the quality of berries. The transcript abundance of DEGs involved in carbohydrate catabolism, carboxylic acid biosynthesis, pyruvate metabolism, glycolysis, organic acid metabolism, phosphoribosyl phosphate biosynthesis, glutamine amino acid metabolism, etc. changed during berry development (from half-véraison to mature). These had large differences in the degree of significant enrichment in different regions, suggesting that genes associated with GO categories may be the main factor in response to environmental changes and mediating differences in berry quality formation (Figure S7). Previous studies have looked at the transcription status of mature grapes in eastern and western China. The term phenylpropane was highly enriched between the two regions [52,53]. As in the current study, phenylalanine metabolism, glycolysis/gluconeogenesis pathway, and glyoxylate were significantly enriched in the LX, PL, RZ, and ZZ regions. The flavonoid biosynthetic pathways in PL and ZZ regions were significantly enriched (Figure S7, Table S7). Additionally, GO categories such as amino acid biosynthesis, plant–pathogen interactions, ribosomes, and MAPK signaling pathway plants (Table S5) deserve further study. Studies on regulating ribosomal proteins have indicated that changes in transcriptome during maturation have led to changes in protein synthesis. This acts as an effective buffer for berries in extreme environments and maintains primary metabolic homeostasis [54,55]. Through further experiments, it is possible to determine the specific effects of environmental factors on berry quality formation. These biological pathways may represent the core transcriptome of berry quality development in different ecological environments.

## 4. Materials and Methods

### 4.1. Plant Material and Environment Condition of Sampling Regions

Grapevine (*Vitis vinifera*) ‘Cabernet Sauvignon’ was used in this study. All plant material was cultivated in an east-west orientation and managed in the same viticultural practices (including north-south direction, single fence, 5 years old grape, pruning system, fertilization, and crown management), in 2020 harvested. Collected grape berries when 50% of the berry skin color was completed as the sample at the half-véraison [56]. The berries were collected at the mature stage when the berry skin coloring was completed and TSS or titratable acidity tended to be stable. Three biological replicates were collected for each sample, each with about 20 berries randomly collected from three trees. All samples were immediately frozen in liquid nitrogen and stored at −80 °C for the following analysis.

The environmental conditions (Table S1) were obtained from the website of the National Centers for Environmental Information (NOAA) (https://www.ncei.noaa.gov/, accessed on 1 October 2020) based on latitude and longitude of sampling regions [57].

### 4.2. Determination of Berry Quality Indices

Berry weight was measured through an analytical balance. TSS of berries were measured using a portable hand-held dialyzer (PAL-1, Japan), and the titratable acidity was titrated with 0.1 mol/L NaOH. Total phenols of the berry were measured using the Folin-Ciocalteu method [58] with some modifications. In a 2 mL Eppendorf tube, 0.44 mL distilled water, 0.06 mL grape pulp extract, 0.25 mL Folin-Ciocalteu (Sigma-Aldrich, Taufkirchen, Germany) reagent, and 0.25 mL sodium carbonate were added and mixed. Then, the samples were held in a dark place at room temperature for an hour. Measured the absorbance of samples at 765 nm with multi-detection microplate reader (CYTATION3, BioTek, Winooski, VT, USA). Gallic acid (Sigma-Aldrich Trading Co., Ltd., Shanghai, China) was used as the standard for drawing the calibration curve to calculate the total phenolic concentration of samples. Anthocyanins were extracted using the method previously described by Zhang et al. and determined using the pH difference method [59]. Three replicates were measured for all indices.

### 4.3. Determination of Sugar, Organic and Phenolic Acids by HPLC

Sugar components mainly glucose and fructose were determined by HPLC (Agilent, Santa Clara, CA, USA, Waters, Milford, MA, USA) according to a method previously reported with some modifications [60]. Three replicates were measured by HPLC. In a 2 mL Eppendorf tube, 0.50 g of berries of each sample were taken and mixed with 1.5 mL 80% ethanol and put in a water bath at 80 °C for 30 min. The samples were centrifuged at 12,000 rpm for 10 min, and then the extract was filtered through a 0.22 μm water filter for injection. The chromatographic conditions for the determination were as follows: Prevail Carbohydrate ES 5μ column (100 mm × 4.6 mm, 5 μm); mobile phase: acetonitrile: water (80%: 20%); column temperature: 50 °C; flow rate: 1.0 mL/min; injection volume: 20 μL. Sugar standards (fructose and glucose) were purchased from Shanghai Macklin Biochemical Technology Co., Ltd. (Shanghai, China).

Organic acid components were determined by HPLC (Agilent, USA, Waters) according to a method previously reported with some modifications [60]. Three replicates were measured by HPLC. The aforementioned extraction method is identical to the extraction of sugar. The chromatographic conditions for the determination were as follows: column. Discovery C18 column (25 cm × 4.6 mm, 5 m); mobile phase: 50 mM K_2_HPO_4_ solution (pH adjusted to 2.4 with phosphoric acid); column temperature 30 °C; flow rate 0.5 mL/min; injection volume 20 μL, detection wavelength was 210 nm. Organic acid standards (tartaric acid, malic acid, and citric acid) were purchased from Shanghai Macklin Biochemical Technology Co., Ltd. (Shanghai, China).

Phenolic components were determined by HPLC with three replicates [61]. 1.00 g of each sample was mixed with 10 mL of 80% methanol and then ultrasonically extracted for 20 min. The extracts were stored in a water bath shaker at 25 °C for 12 h, filtered with a 0.45 µm filter, and stored at −40 °C for the next analysis. The chromatographic conditions were set as follows: HPLC 1260 (Agilent, USA, Waters Xterra RP18 (100 9 4.6 mm, 3.5 μm) column), mobile phase A: water-formic acid (0.2%), B: acetonitrile- water-formic acid 0.2% (80: 20), flow rate 0.6 mL/min, column temperature at 30 °C, injection volume: 20 μL. Phenolic standards (epicatechin, epigallocatechin, epicatechin gallate, catechin, proanthocyanidins B1, and proanthocyanidins B2) were purchased from Sigma-Aldrich Trading Co., Ltd., (Shanghai, China). All solvents were of pure analytical quality or HPLC grade, purchased from Merck, Darmstadt, Germany.

### 4.4. Determination of Anthocyanin Components by LC-MS

Anthocyanin components were determined by LC-MS with three replicates [59]. 1.00 g of berry peel was mixed with 0.1% hydrochloric acid-methanol. Extraction was completed with an ultrasonic bath for 20 min and was centrifuged at 4 °C for 15 min. The liquid was filtered through a 0.22 mm organic column. The anthocyanins were analyzed by LC-MS (G2-XS QT, USA, Waters), using a chromatographic column: 2.1 × 100 mm ACQUITY UPLC BEH C18 column; flow rate: 0.4 mL/min; injection volume: 2 µL. Buffer A was 0.1% formic acid in the water, and buffer B was 0.1% formic acid-acetonitrile solution. Anthocyanin standards (malvidin, delphinidin, petunidin, cyanidin, and peonidin) were purchased from Sigma-Aldrich Trading Co., Ltd., (Shanghai, China).

### 4.5. RNA Extraction, cDNA Library Construction, and Transcriptome Sequencing

Total RNA of all samples was extracted using the modified Hexadecyltrimethylammonium Bromide (CTAB) method [62]. DNase (Takara, Beijing, China) was added to digest DNA and remove interference. RNA concentration was measured using a NanoDrop 2000 spectrophotometer (Thermo Scientific, Wilmington, DE, USA) and RNA quality was assessed on a 1% denatured agarose gel. The RNA library preparation kit was used to generate purified mRNA and library construction for Illumina (New England BioLbs, Ipswich, MA, USA). Finally, the PCR products were purified (AMPure XP system) and library quality was assessed on the Agilent Bioanalyzer 2100 system. Then, the qualified cDNA library was used for RNA-seq analysis. Two biological replicates from each sample were used for transcriptome sequencing.

After checking with the library, all samples were sequenced on Illumina Hiseq, and paired-end 150 bp reads were generated. Transcriptome data of ‘Cabernet Sauvignon’ from multiple regions were registered in NCBI (https://dataview.ncbi.nlm.nih.gov/object/PRJNA890029?reviewer=4igniliglls6b7v7803nhos9aq, accessed on 13 October 2022) and the accession number of RNA-seq data is PRJNA890029.

### 4.6. RNA-Seq Analysis

Raw sequences were filtered to remove the adaptor sequence, low-quality reads, and short reads. The resulting sets of high-quality clean reads were used for transcriptome analysis. High-quality clean reads were mapped to the grape reference genome (*Vitis vinifera* cultivar: Muscat Hamburg (wine grape)) (https://www.ncbi.nlm.nih.gov/bioproject/671671, accessed on 18 December 2020) to obtain uni-genes using Hisat2 v2.0.5 [63]. The expressed values of all genes were calculated and normalized according to fragments per kilobase of transcript per million mapped reads (FPKM). Differentially expressed genes (DEGs) were recruited by |log2 (fold change)| ≥ 1 and padj ≤ 0.05. The GO (http://www.geneontology.org/) and KEGG database (http://www.genome.jp/kegg/) were used for all genes enrichment analysis, and the function defining padj ≤ 0.05 was significantly enriched. The Up-set Plot in TBtools [64] (https://github.com/CJ-Chen/TBtools/releases, accessed on 5 March 2021) was used to perform statistical analysis on the common or specific DEGs. The Graphics of Circos software in TBtools was used for the analysis of the gene expression and the distribution of DEGs on the chromosomes.

### 4.7. Quantitative Real-Time PCR Analysis

Several DEGs were selected for quantitative real-time PCR (qRT-PCR) analysis using the designed specific primers by NCBI Primer Blast (https://www.ncbi.nlm.nih.gov/tools/primer-blast/index.cgi?LINK_LOC=BlastHome, accessed on 24 September 2020) (Table S9). First-strand complementary DNA (cDNA) was synthesized from RNA samples in three biological replicates using a Hieff First Strand cDNA Synthesis Super Mix (11103ES70, YEASEN, Shanghai, China) according to the manufacturer’s instructions. qRT-PCR was performed using an Ultra SYBR Mix kit (YEASEN, Shanghai, China). Actin (GenBank Accession number AM486252.2) was used as an internal standard for normalization. Three technical replicates were performed for each sample. Relative expression levels of uni-genes were detected using the 2^−ΔΔCT^ method [65]. The experiments were repeated at least two times with similar results, although the representative data from one repetition were shown.

### 4.8. Statistical Analysis

The data were presented as mean ± standard deviation (SD) (n = 3). Statistical analysis of variance (ANOVA) was performed using SPSS 17.0 (SPSS, Inc., Chicago, IL, USA) with Duncan’s multiple range test at *p* < 0.05. The correlation between environmental conditions and quality characteristics was analyzed using Pearson correlation coefficient. The Origin Pro 9 (Origin Inc., Northampton, MA, USA) was used to produce the figures.

## 5. Conclusions

A comprehensive analysis herein of the quality of ‘Cabernet Sauvignon’ in different regions revealed that regional diversity provided a significant effect in forming the unique quality of ‘Cabernet Sauvignon’. This research contributed to the understanding of the interaction of grape quality with environmental conditions and the effect of the latter on the formation of berry metabolites. Among them, phenols, anthocyanins, and titratable acids were the most sensitive indicators of environmental factors and the main factors making up the regionality of berry quality. It should be noted that the change in titrating acids and anthocyanins from half-véraison to maturity varies greatly between regions. Additionally, RNA-seq has proved to be a valuable tool for monitoring changes in grape quality dependent on the regional environment, improving the interpretation of plasticity in the quality physiological composition of grapes depending on the environment. The information generated from this study could contribute to the elaboration of viticultural practices aimed at making better use of native varieties to develop wines with regional characteristics.

## Data Availability

The datasets generated analyzed during the current study are available in the NCBI repository, (https://dataview.ncbi.nlm.nih.gov/object/PRJNA890029?reviewer=4igniliglls6b7v7803nhos9aq, accessed on 13 October 2022).

## Supplementary Materials

The supporting information can be downloaded at: https://www.mdpi.com/article/10.3390/ijms24054670/s1.

## Abbreviations

C.V	coefficient of variation
DZ	Dezhou region
LX	Laixi region
PL	Penglai region
RZ	Rizhao region
RS	Rushan region
TA	Tai’an region
ZZ	Zaozhuang region
qRT-PCR	Quantitative real-time PCR
PCA	Principal Component Analysis
NCBI	National center for biotechnology information
KEGG	Kyoto Encyclopedia of Genes and Genomes
HPLC	High-performance liquid chromatographic
GO	Gene Ontology
FPKM	Fragments Per Kilobase of transcript per Million mapped reads
DEGs	Differentially expressed genes
CTAB	Hexadecyltrimethylammonium Bromide
TSS	Total Soluble Solids
