# Supplementary material for: Assessment of ‘Cabernet Sauvignon’ Grape Quality Half-Véraison to Maturity for Grapevines Grown in Different Regions
Source: Int J Mol Sci. 2023 Feb 28;24(5):4670. doi: 10.3390/ijms24054670 (PMC10002954; doi:10.3390/ijms24054670)
Supplement: Supplementary file 1 [file ijms-24-04670-s001.zip › Supplementary figures.pdf]

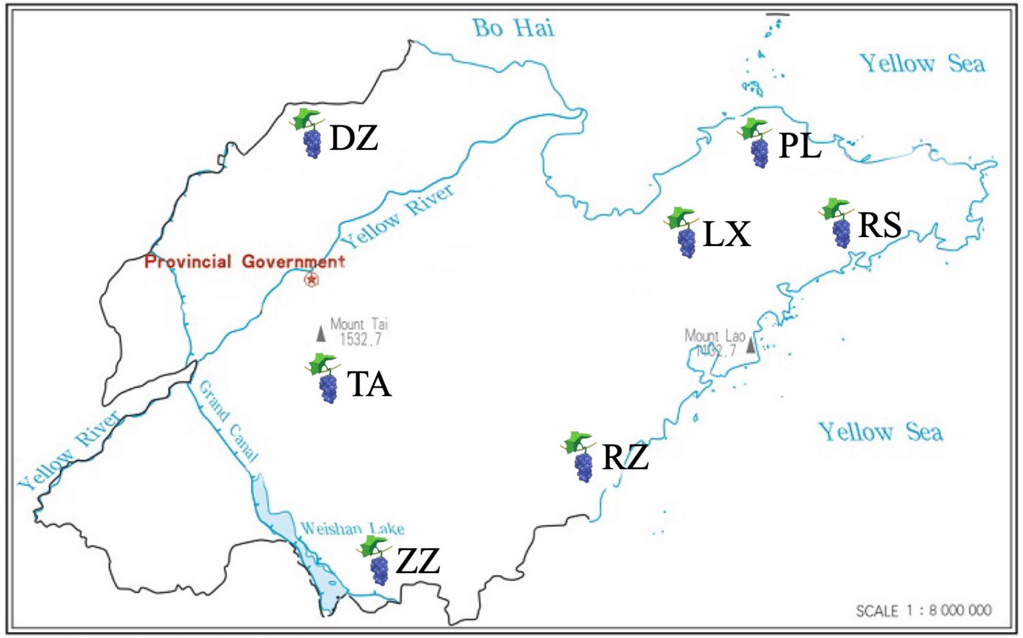

Figure S1 Sample collection distribution map. Seven regions were included Dezhou (DZ), Laixi (LX), Penglai (PL), Rizhao (RZ), Rushan (RS), Tai'an (TA), and Zaozhuang (ZZ).

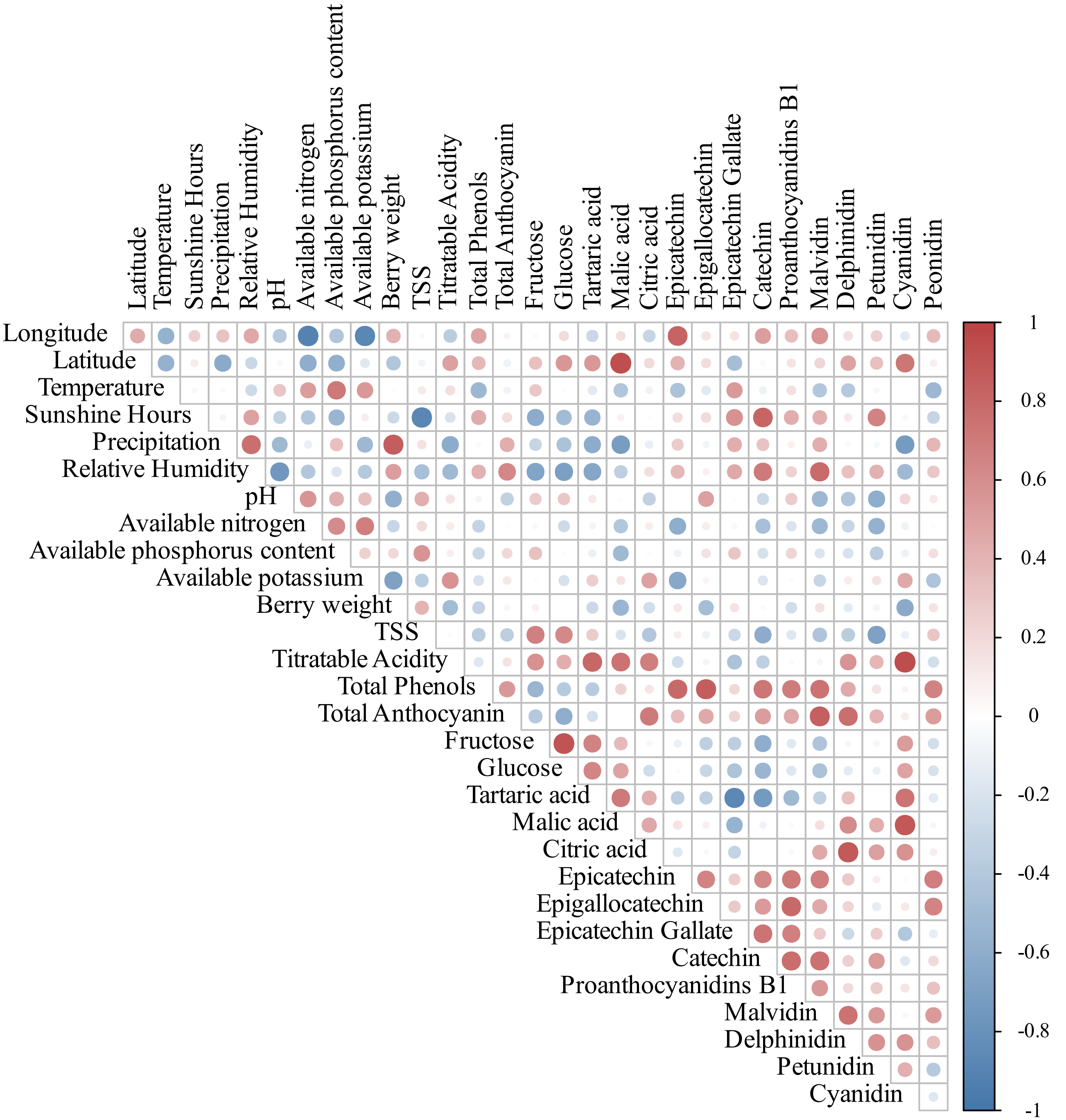

Figure S2 Correlation analysis of environmental factors and quality characteristics

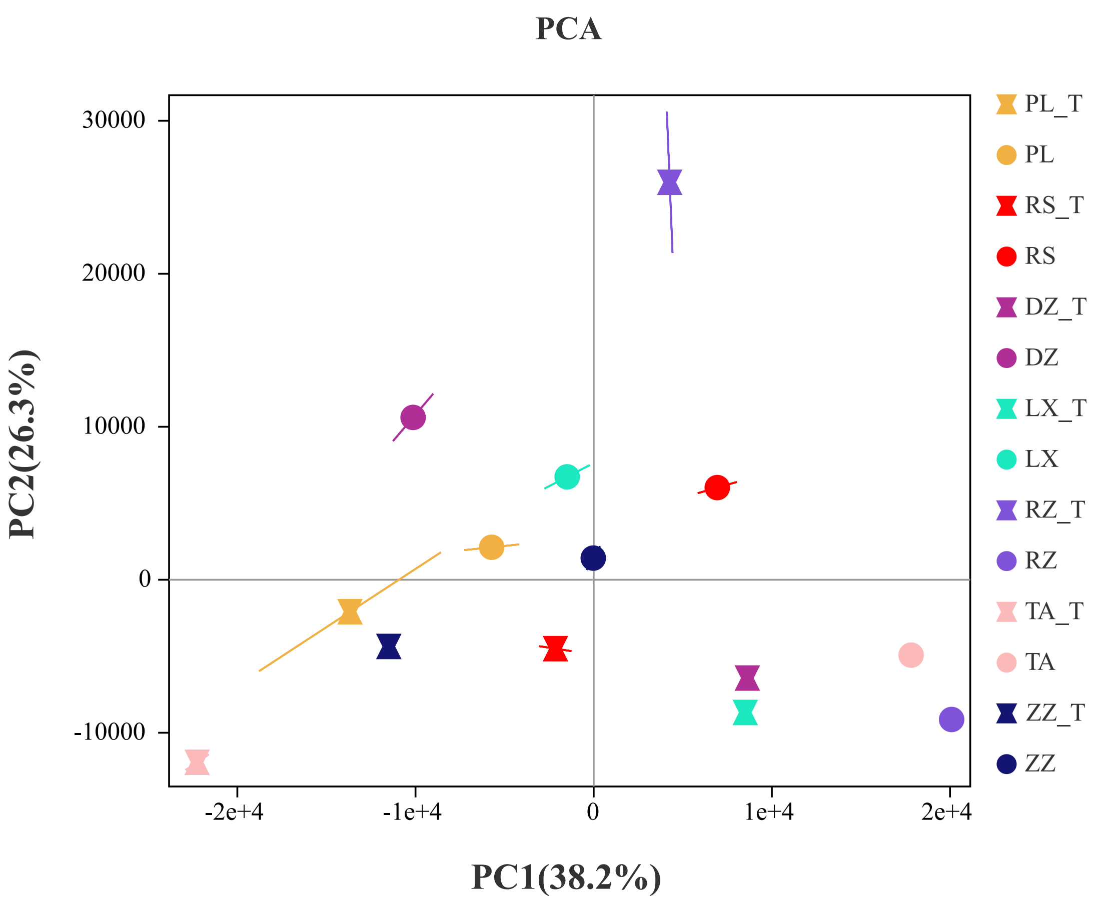

Figure S3 PCA across ecoregions. Seven regions were included Dezhou (DZ), Laixi (LX), Penglai (PL), Rizhao (RZ), Rushan (RS), Tai'an (TA), and Zaozhuang (ZZ). In this figure T means half-véraison.

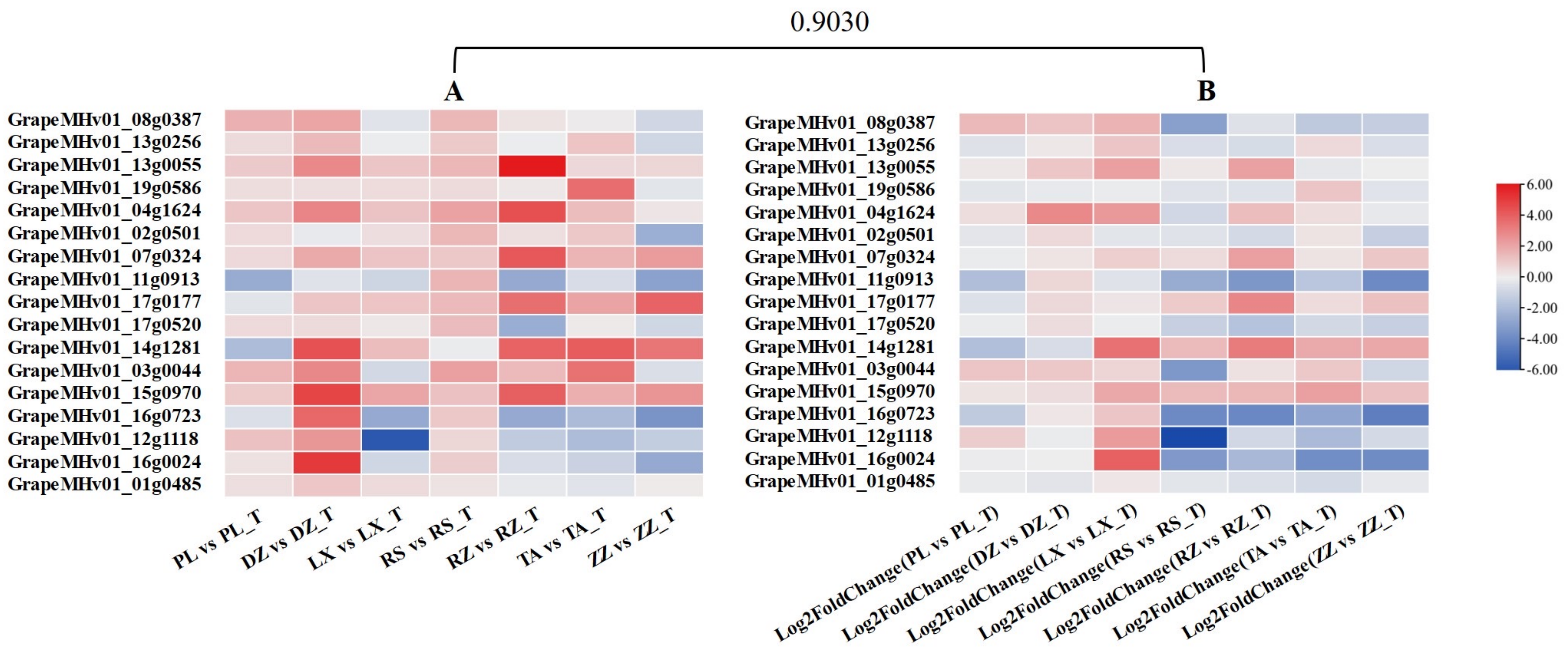

Figure S4 Comparing the RNA-seq data of the selected DEGs with the qRT-PCR results. A: qRT-PCR data, these values values were mean ± SD (n = 3), B: Validation RNA-seq data, the relative expression ratio of each DEGs was expressed as log2(FoldChange). Seven regions were included Dezhou (DZ), Laixi (LX), Penglai (PL), Rizhao (RZ), Rushan (RS), Tai'an (TA), and Zaozhuang (ZZ). In this figure 'T' means half-véraison.

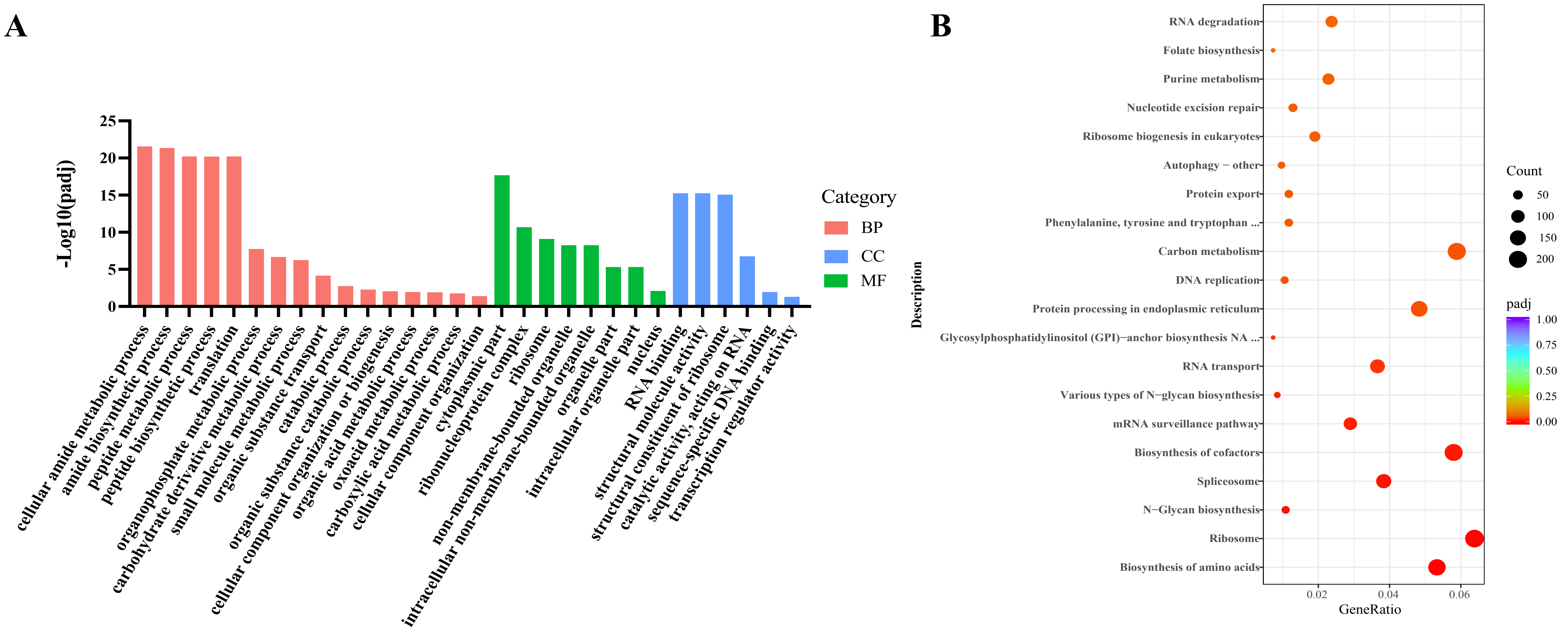

Figure S5 Histogram shows that the number of DEGs between the comparative combinations of different regions at half-véraison. B: Dot plot.

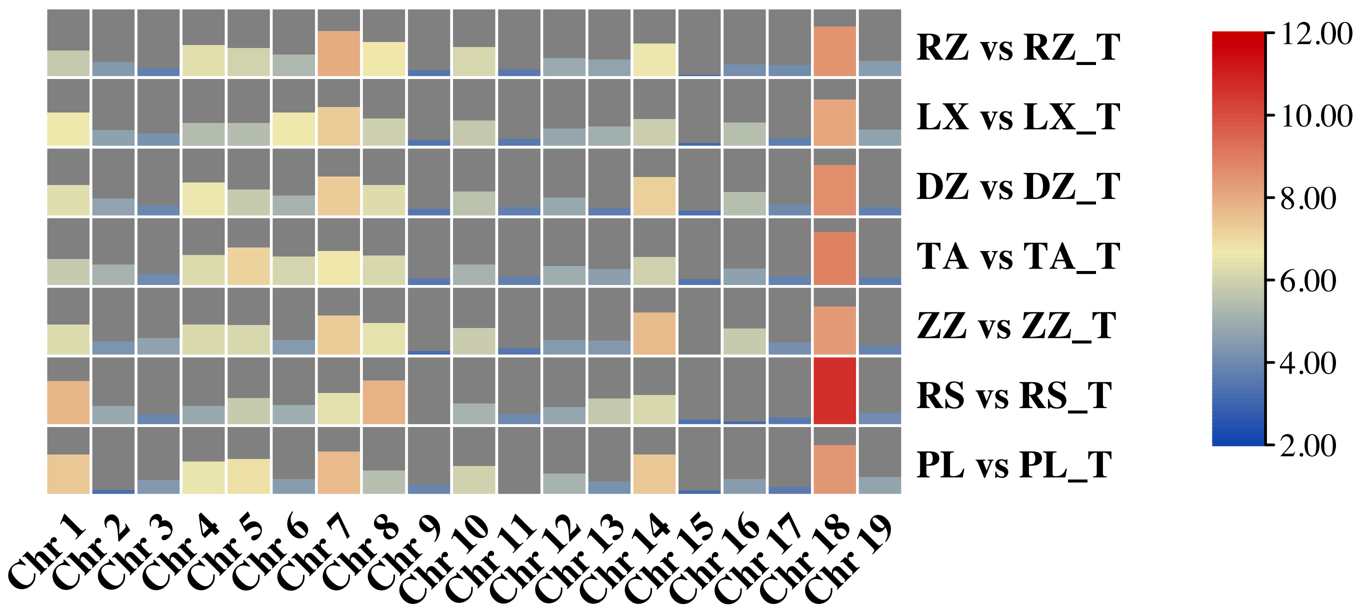

Figure S6 Heatmap representing the number of DEGs enriched on 19 chromosomes. Seven regions were included Dezhou (DZ), Laixi (LX), Penglai(PL), Rizhao (RZ), Rushan (RS), Tai'an (TA), and Zaozhuang (ZZ). In this figure T means half-véraison.

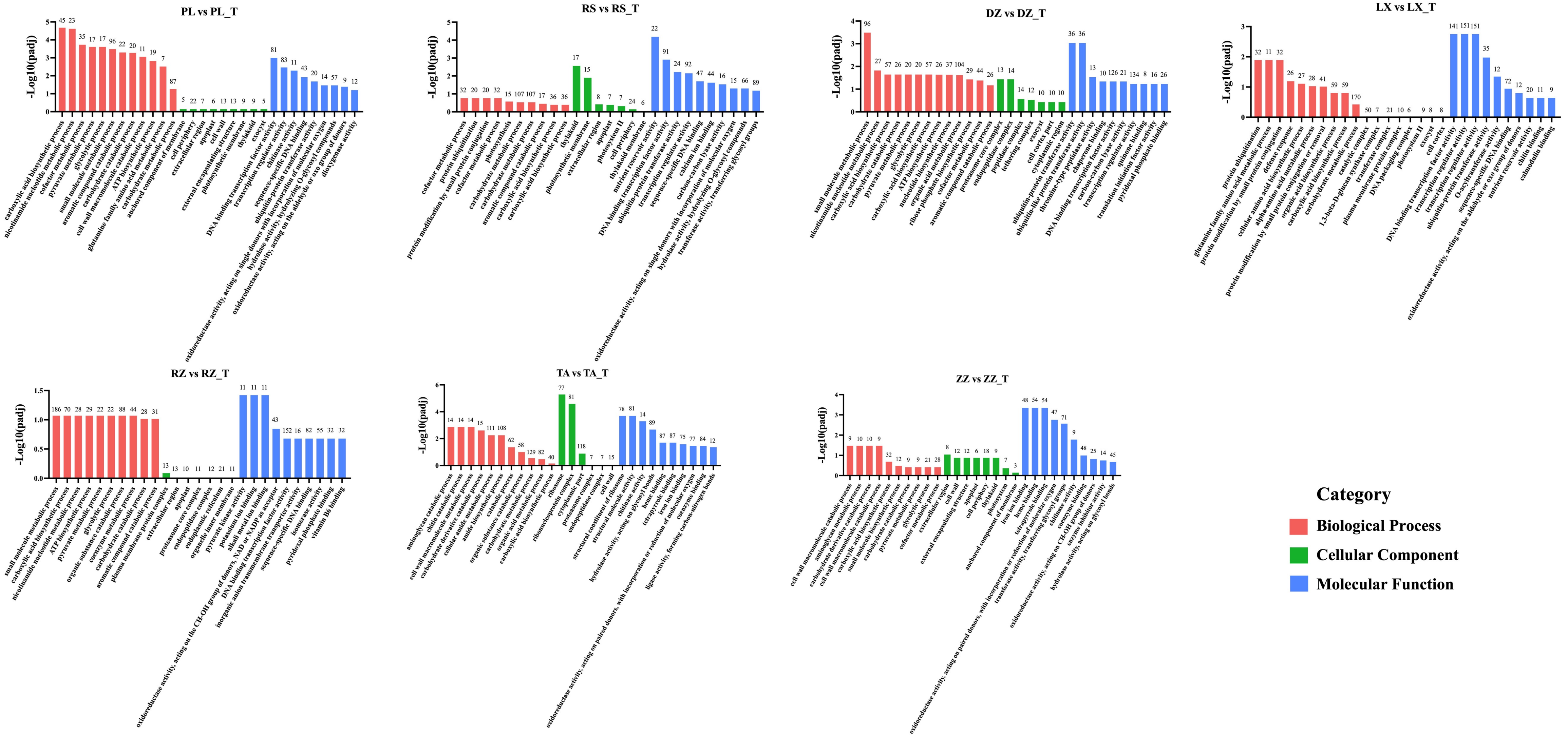

Figure S7 The Go enrichment classification of DEGs. X-axis represents GO terms, and Y-axis represents indigenous level of GO categories. Seven regions were included Dezhou (DZ), Laixi (LX), Penglai (PL), Rizhao (RZ), Rushan (RS), Tai'an (TA), and Zaozhuang (ZZ). In this figure T means half-véraison.
